# DarkCideS 1.0, a global database for bats in karsts and caves

**DOI:** 10.1038/s41597-022-01234-4

**Published:** 2022-04-05

**Authors:** Krizler C. Tanalgo, John Aries G. Tabora, Hernani Fernandes Magalhães de Oliveira, Danny Haelewaters, Chad T. Beranek, Aída Otálora-Ardila, Enrico Bernard, Fernando Gonçalves, Alan Eriksson, Melissa Donnelly, Joel Monzón González, Humberto Fernández Ramos, Alberto Clark Rivas, Paul W. Webala, Stanimira Deleva, Ridha Dalhoumi, Jaycelle Maula, Dennis Lizarro, Luis F. Aguirre, Nils Bouillard, Ma. Niña Regina M. Quibod, Jennifer Barros, Manfredo Alejandro Turcios-Casco, Marcio Martínez, Diego Iván Ordoñez-Mazier, José Alejandro Soler Orellana, Eduardo J. Ordoñez-Trejo, Danny Ordoñez, Ada Chornelia, Jian Mei Lu, Chen Xing, Sanjeev Baniya, Renata L. Muylaert, Leonardo Henrique Dias-Silva, Nittaya Ruadreo, Alice Catherine Hughes

**Affiliations:** 1 Center for Integrative Conservation, Xishuangbanna Tropical Botanical Garden, and the Center for Conservation Biology, Core Botanical Gardens, Chinese Academy of Sciences, Yunnan, P.R. China; 2 International College of the Chinese Academy of Sciences, Beijing, P.R. China; 3 Ecology and Conservation Research Lab (Eco/Con Lab), Department of Biological Sciences, College of Science and Mathematics, University of Southern Mindanao, Kabacan, North Cotabato, Philippines; 4 Zukunftskolleg and the Center for Advanced Study of Collective Behaviour, University of Konstanz, Universitätsstrasse 10, Baden-Württemberg, Konstanz, Germany; 5 School of Environmental Science and Management, University of the Philippines, Los Banos, Laguna Philippines; 6 Department of Zoology, Federal University of Paraná, Curitiba, PR Brazil; 7 Research Group Mycology, Department of Biology, Ghent University, 9000 Ghent, Belgium; 8 Operation Wallacea Ltd, Wallace House, Old Bolingbroke, Lincolnshire, PE23 4EX United Kingdom; 9 Faculty of Science, University of South Bohemia, 370 05 České Budějovice, Czech Republic; 10 School of Environmental and Life Sciences, Biology Building, University of Newcastle, University Drive, Callaghan, NSW 2308 Australia; 11 FAUNA Research Alliance, PO Box 5092, Kahibah, NSW 2290 Australia; 12 Grupo en Conservación y Manejo de Vida Silvestre, Universidad Nacional de Colombia, Bogotá, Colombia; 13 Laboratório de Ciência Aplicada à Conservação da Biodiversidade, Department of Zoology, Universidade Federal de Pernambuco (UFPE), Pernambuco, Brazil; 14 Department of Biodiversity, Institute of Bioscience, Universidade Estadual Paulista (UNESP), Rio Claro, São Paulo, Brazil; 15 Conservation Science Group, Department of Zoology, University of Cambridge, Cambridge, UK; 16 Programa de Pós‐Graduação em Ecologia e Conservação, Instituto de Biociências, Universidade Federal de Mato Grosso do Sul, Campo Grande, Brazil; 17 Proyecto CUBABAT, Calle América #6503 (Altos) e/ Jáuregui y Santa Isabel, 40100 Matanzas, Cuba; 18 Fundación “Antonio Núñez Jiménez” de la Naturaleza y el Hombre, Calle 5ta B, No. 6611 e/ 66 y 70, Miramar, Playa, La Habana, Cuba; 19 Sociedad Espeleológica de Cuba (SEC), Calle 9na. #8402 e/ 84 y 84ª. Playa, La Habana, Cuba; 20 Department of Forestry and Wildlife Management, Maasai Mara University, Narok, Kenya; 21 Sede del Sur, Universidad de Costa Rica, 4000 Alamedas, Golfito, 60701 Costa Rica; 22 National Museum of Natural History-Bulgarian Academy of Sciences, Sofia, Bulgaria; 23 Laboratoire de Biosurveillance de l’Environnement, Faculté des Sciences de Bizerte, Université de Carthage, 7021 Zarzouna, Tunisia; 24 Department of Biology, Southern Luzon State University, Lucban, Quezon, Philippines; 25 Centro de Investigación de Recursos Acuáticos, Universidad Autónoma del Beni “José Ballivián” (CIRA-UABJB). Campus “Dr. Hernán Melgar Justiniano”, Santísima Trinidad, Beni, Bolivia; 26 Programa para la Conservación de los Murciélagos de Bolivia. Cochabamba y Beni, Beni, Bolivia; 27 Centro de Biodiversidad y Genética, Universidad Mayor de San Simón, Casilla 538, Cochabamba, Bolivia; 28 Barbastella Echology, Gentpoortstraat 50, 9800 Deinze, Belgium; 29 Museum of Natural History of the University of the Philippines, Los Banos, Laguna, Philippines; 30 Asociación para la Sostenibilidad e Investigación Científica en Honduras (ASICH). Barrio La Granja, entre 28 y 29 calle, C. P. 504. Comayagüela M.D.C. Francisco Morazán, Tegucigalpa, Honduras; 31 School of Zoology, Faculty of Life sciences, Tel Aviv University, Tel Aviv, Israel; 32 National Centre for Biological Sciences (NCBS), Bangalore, India; 33 Molecular Epidemiology and Public Health Laboratory, Hopkirk Research Institute, Massey University, Palmerston North, New Zealand; 34 Laboratório de Mastozoologia do Departamento de Biologia Animal da Universidade Federal de Viçosa, Minas Gerais, Viçosa, Brasil; 35 Division of Biological Sciences, Faculty of Science, Prince of Songkla University, Hat Yai, Songkhla, Thailand; 36 School of Biological Sciences, The University of Hong Kong, Hong Kong SAR, China

**Keywords:** Biodiversity, Macroecology

## Abstract

Understanding biodiversity patterns as well as drivers of population declines, and range losses provides crucial baselines for monitoring and conservation. However, the information needed to evaluate such trends remains unstandardised and sparsely available for many taxonomic groups and habitats, including the cave-dwelling bats and cave ecosystems. We developed the DarkCideS 1.0 (https://darkcides.org/), a global database of bat caves and species synthesised from publicly available information and datasets. The DarkCideS 1.0 is by far the largest database for cave-dwelling bats, which contains information for geographical location, ecological status, species traits, and parasites and hyperparasites for 679 bat species are known to occur in caves or use caves in part of their life histories. The database currently contains 6746 georeferenced occurrences for 402 cave-dwelling bat species from 2002 cave sites in 46 countries and 12 terrestrial biomes. The database has been developed to be collaborative and open-access, allowing continuous data-sharing among the community of bat researchers and conservation biologists to advance bat research and comparative monitoring and prioritisation for conservation.

## Abstract (summary)

**Table:** 

Measurement(s)	spatial region
Technology Type(s)	occurrent
Sample Characteristic - Organism	Mammalia • Chiroptera sp. BOLD:AAA2524
Sample Characteristic - Environment	cave system • karst cave
Sample Characteristic - Location	Global

## Background & Summary

Human civilization has left its footprint on every part of the planet, in the process driving what is frequently referred to as the sixth mass extinction^1,2^. Conservation prioritisation requires a rigorous assessment of vulnerable species as well as their habitats to develop priorities for conservation. Biodiversity data integration and synthesis are significant empirical steps to identify priorities in strategically using the limited funds allocated to conservation^3^. However, the data needed to develop such priorities with rigour are often lacking. The diversity and distribution of a subset of terrestrial vertebrates have become an umbrella for taxonomic and spatial conservation, despite the known biases present in popular open datasets^4,5^. Efforts to mitigate extinction risks or protect key habitats often disproportionately focus on particular taxa, ecosystems, or regions^6,7^. This approach neglects many other equally important species and their habitats and compromises the maintenance of ecosystem services provided by diverse functional groups^8,9^.

Cave ecosystems are critical for bats, with around half of all bat species reliant on caves, with a high rate of endemism^10,11^. Of the more than 1400 known extant bat species distributed across almost all terrestrial habitats around the globe, at least 679 species are known to be cave-dwelling^11–13^. Many of these species occur in biodiversity hotspots that are threatened by varying anthropogenic and natural threats^13,14^. Caves are important habitats for bats and other unique species but are nonetheless threatened and in need of urgent conservation^10^. Despite hosting high endemism, cave ecosystems receive little attention in terms of fund allocation and appropriate priorities for scientific studies and conservation compared to their surface counterparts such as agricultural and forest ecosystems^10,13,15–18^. Cave taxa are adapted to light-limited underground environments and most of them are dependent on mobile species such as bats to transport organic nutrients into these environments^19–21^. Bats are keystone species in karst ecosystems and ideal cave conservation surrogates, delivering vital energy sources into caves as they regularly forage from outside ecosystems^22^. Nevertheless, conservation attention towards cave-dwelling bats remains limited compared to other mammalian taxa. Thus, there is an urgent need for better data to develop effective conservation strategies for bats^13^.

Effective conservation decision-making relies on the accuracy and precision of the data used to design present and future management strategies^5,7^. Identifying priority caves for conservation requires an understanding of species diversity, endemism patterns, interactions with other organisms, and threats within and outside these systems^17,23^. Additionally, while numerous organisations and collaborative efforts aim to database bat distributions, comprehensive and specific datasets for cave-dwelling bats, including their distributions and ecological traits, are currently lacking. Large databases for species distributions such as the Global Biodiversity Information Facility (GBIF) exist and openly provide distribution data for bats. However, due to the enormous amount of information within these databases, it is challenging to selectively evaluate data for specific ecosystems such as caves, and thus more specialist datasets are needed to facilitate appropriate habitat-based prioritisation.

**Fig. 1:**
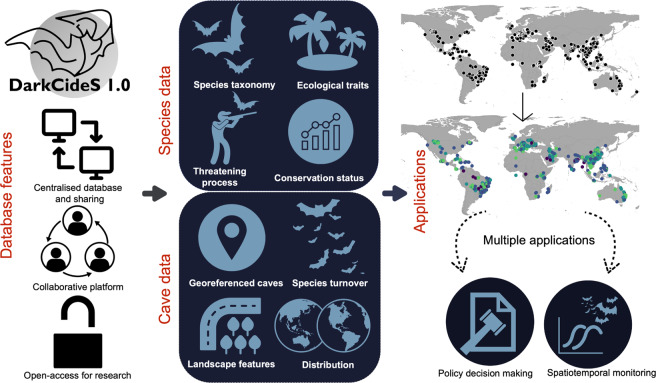
A schematic diagram showing the features, contents, and potential applications of the DarkCideS 1.0 database. The database is a centralised, collaborative, and open-access platform that contains information on cave-dwelling bat species and their distribution.

To address this knowledge gap, we created DarkcideS 1.0 (https://darkcides.org/), a global database for bats in karsts and caves, to advance global bat cave vulnerability and conservation mapping initiatives. The creation of the dataset primarily aims to map and digitise the distribution of cave-dwelling bats to facilitate the assessment of their vulnerability to landscape threats. DarkCideS 1.0 represents a publicly available database of cave-dwelling bats across time and space, including their estimated population (e.g., counts), geographical distribution (latitude and longitude), ecological traits, levels of endemism, conservation status, and threatening processes. The purpose of the DarkCideS 1.0 initiative is to centralise and develop an open-access platform for information exchange among bat researchers and conservation biologists to advance the development of targeted conservation measures and macroecological studies (Fig. 1). Potential applications of the database include assessing species conservation status and extinction risks; understanding drivers of extinction, cave conditions, and landscape threats; accurately developing species distribution models; and determining long-term cave conservation priorities at regional to global scales.

## Methods

The DarkCideS database was initially conceptualised and developed by KCT, JAG, and ACH as part of the “Global Bat Cave Vulnerability and Conservation Mapping Initiative” in 2014, and later with the “Mapping Karst Biodiversity in Yunnan” and the “Southeast Asian Atlas of Biodiversity” projects. The initiative includes developing tools and methods (e.g., the Bat Cave Vulnerability Index^14^) and synthesis (e.g., the global bat cave vulnerability assessment^11^) to identify conservation priorities and important bat caves in the tropics. Since 2019, the initiative has expanded and potential collaborators and contributors were invited through scientific conferences (Association for Tropical Biology and Conservation 2018, International Bat Research Conference 2019), social media platforms, and personal correspondences. At present, the database has 36 collaborators from twenty countries on six continents with expertise and research interests in bat conservation. Four main datasets for all known cave-dwelling bats were built for the DarkCideS database version 1.0.

### Datasets and compilation for species checklist

The first dataset contains taxonomic checklists for all extant cave-dwelling bats species extracted from the expert-based International Union for the Conservation Union (IUCN) Red List database version 2020-1 (Table 1). We screened and included all bat species that were reported to use, roost in, or aggregate in “Caves”, “Underground”, and “Karsts” habitats in any part of their life histories. We also scanned major publicly available bat cave databases from expeditions such as “Bats in China” (http://www.bio.bris.ac.uk/research/bats/China/) and UNEP-EUROBATS (https://www.eurobats.org/) for European bats^24^ for additional information and datasets. In addition, the first dataset contains species ecological traits, distribution range, and threatening processes (Table 1).

**Table 1:** DarkCideS 1.0 includes key traits for all living cave-dwelling bat species (N = 679). General metadata for traits included in the current version of the database: habitat preference, ecological status, feeding groups, geographical range, island endemism, geopolitical endemism, distribution range, biogeographical breadth, generation length, body mass, and threatening process.

Trait category	Trait (Data name)	Variable type	Data filters	N species	Sources
Habitat preference	Forest	Binomial	Yes = 1, No = 0	586	IUCN Red List database
	Savanna			140	
	Desert			45	
	Urban			16	
	Underground			523	
	Wetlands			56	
Ecological status and distribution	Population.status	Nominal	Decreasing	150	
			Stable	161	
			Increasing	6	
			Unknown	362	
	Conservation.status		Data.Deficient	83	
			Least.Concern	452	
			Near.Threatened	54	
			Vulnerable	54	
			Endangered	25	
			Critically.Endangered	11	
	Geopolitical.endemism		Non.Endemic	459	
			Endemic	220	
	Island.endemism		Island.Endemic	159	Phylacine 1.2
			Mainland	520	
	Biogeographic.breadth		Afrotropical	102	
			Indomalayan	184	
			Austral-Oceania	49	
			Neotropical	173	
			Palearactic	85	
			Neactic	18	
			Cosmopolitan	68	
Feeding groups	Feeding.groups		Carnivore	553	EltonTraits 1.0
			Frugi-nectarivore	60	
			Omnivore	66	
Geographical range	Island.endemism	Nominal	Islandic	160	Phylacine 1.2
			Non-islandic	521	
	Current.range	Continuous	N/A	679	Phylacine 1.2
	Natural.range	Continuous	N/A	679	
Biological traits	Generation.length	Continuous	N/A	679	Pacifi *et al*. (2013)
	Body.mass (grams)	Continuous	N/A	679	Phylacine 1.2
Direct threats	Mining.quarrying	Binomial	Yes = 1, No = 0	155	IUCN Red List database
	Sacred.activities			11	
	Tourism.caving			226	
	Guano.extraction			69	
	Vandalism			106	
	Nest.harvesting			5	
	Hunting.bushmeat			109	
	Intensional.killings			48	
	Gating			7	
	Scientific.research			7	
Indirect threats	Agricultural.conversion			155	
	Urbanisation			76	
	Deforestation			284	
	Pollution			65	
	Road.kills			12	
Natural threats	Disease.parasites			5	
	Invasive.species			21	
	Fires			36	
	Drought			9	
	Extreme.cold			1	
	Storm			17	

**Fig. 2:**
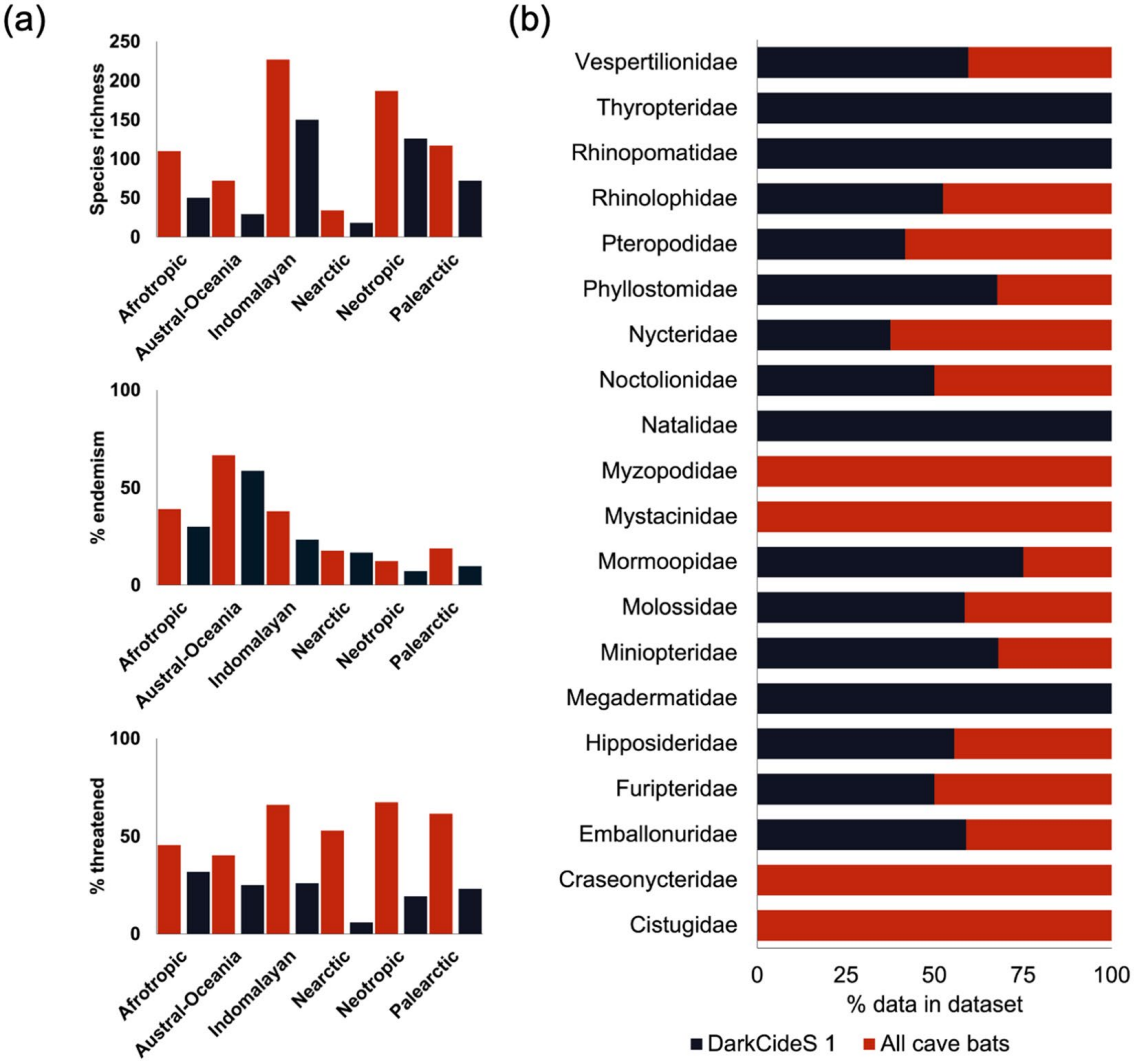
Percentage of species data completeness according to biogeographical realm (**a**) and family-level (**b**) between IUCN estimates (red bars) and sampled caves from DarkCideS 1.0 (black bars) species richness, the proportion of endemism, and proportion of threatened species worldwide.

Information per species was pooled from the IUCN Red List versions 2020-1^25^. Species taxonomy was then curated and updated (e.g., synonyms or merged species) using the nomenclature from Simmons and Cirranello^12^. The “checklist for global cave-dwelling bats” derived from the IUCN Red List includes 679 species. Meanwhile, the DarkCideS 1.0 dataset contains occurrence data for 402 species from 16 families representing 59% of all cave-dwelling species^11^ (Fig. 2). We found a marginally significant relationship between the species richness and proportion of threatened species between the IUCN-based global cave-dwelling bat and DarkCideS datasets (Kendall’s *τ* b = 0.60, *P* = 0.07). The highest completeness of sampled species is in the Neotropics (67.38%) and Indomalayan region (66.08%), and the greatest gaps are in Austral-Oceania (40.28%). Highest endemism was recorded in Austral-Oceania (58.62%) (χ^2^ = 227.32, df = 5, P < 0.001) (Fig. 2a). The proportion of threatened species is highest in the Indomalayan region (16%) realm (χ^2^ = 281.18, df = 5, P < 0.01) (Fig. 2a). Most bat families have a coverage of 30 to 60% of species, but four families had all cave-dwelling species in the DarkCideS database, and three smaller families had no species included (Fig. 2b).

### Habitat preference, distribution, ecological status, and traits

We classified species distribution by biogeographical realm (Indomalaya, Austral-Oceania, Afrotropical, Neotropical, Palearctic, and Nearctic) and terrestrial biomes following Olson *et al*.^26^. We described species’ major habitat breadth based on IUCN Level 1 classification https://www.iucnredlist.org/resources/habitat-classification-scheme (Caves, Forests, Savanna, Desert, Urban, Artificial, and Wetlands). Species current conservation status (Data Deficient, Least Concern, Near Threatened, Vulnerable, Endangered, and Critically Endangered) and population trends (e.g., Unknown, Decreasing, Stable, Increasing) were categorised using standard IUCN Red List assessments. Using the same criteria, we categorised species endemism as geopolitically endemic (e.g., country-endemic, and non-endemic) when a species occurs only in a single country or state territory^27^, and island endemism was classified as island-restricted or predominantly mainland^28^. The highest country endemism was in the Eastern Hemisphere with the highest in the Austral-Oceania (40%) region, followed by the Afrotropical (21%), then the Indomalayan region (16%). However, the highest proportion of threatened species was in the Indomalayan region (43%) and the Neotropics (22%) (Fig. 2a).

Furthermore, current geographical ranges were assembled from the Phylacine 1.2 database^28^ based on IUCN species ranges. Three species traits were included: the adult body mass (in grams) per species were derived from Phylacine 1.2^28^ and generation length from Pacifici *et al*.^29^. For trophic groups, we derived diet information from EltonTraits 1.0^30^. We grouped species as frugi-nectarivorous for all species that forage on plant-based resources (e.g., fruits, leaves, and nectars). As species foraging smaller vertebrates (i.e., fish, birds, and rodents) and larger invertebrates are very few, we classified them as carnivores along with insectivorous bats. Species that forage on both resources were grouped into omnivores (Table 1).

### Species threatening process

We identified potential threats for each bat species listed in the checklist using the information from the IUCN Red List assessments (version 2020-1) in addition to threats highlighted in the literature. The IUCN Red List standardised its classification based on Salafsky *et al*.^31^, but we reclassified the threatening process into three key categories: Direct, Indirect, and Natural (Table 1) based on the drivers of threat^10,14,32^. Direct threats (*T*_dir_) refer to the threats or risks that are direct to or in cave systems with immediate and perceivable impacts on populations or behaviour of species. This category includes direct human impacts (e.g., persecution, eviction, and cave closures) and the use of caves for harvesting bats, tourism, religious visits, and mining (minerals or guano). Indirect threats (*T*_ind_) refer to the threats outside cave systems or within cave proximity, of which the impacts to populations are secondary or non-immediate but otherwise detrimental. Examples include deforestation, agriculture, and urbanisation. Lastly, Natural threats (*T*_nat_) refer to threats that are natural in origin, though their frequency may be impacted by human activities, and that may directly or indirectly impact populations, such as diseases (e.g., White-nose syndrome) and climate-driven risks (e.g., drought, extreme cold) (Table 1).

### Bat cave georeferencing

**Fig. 3:**
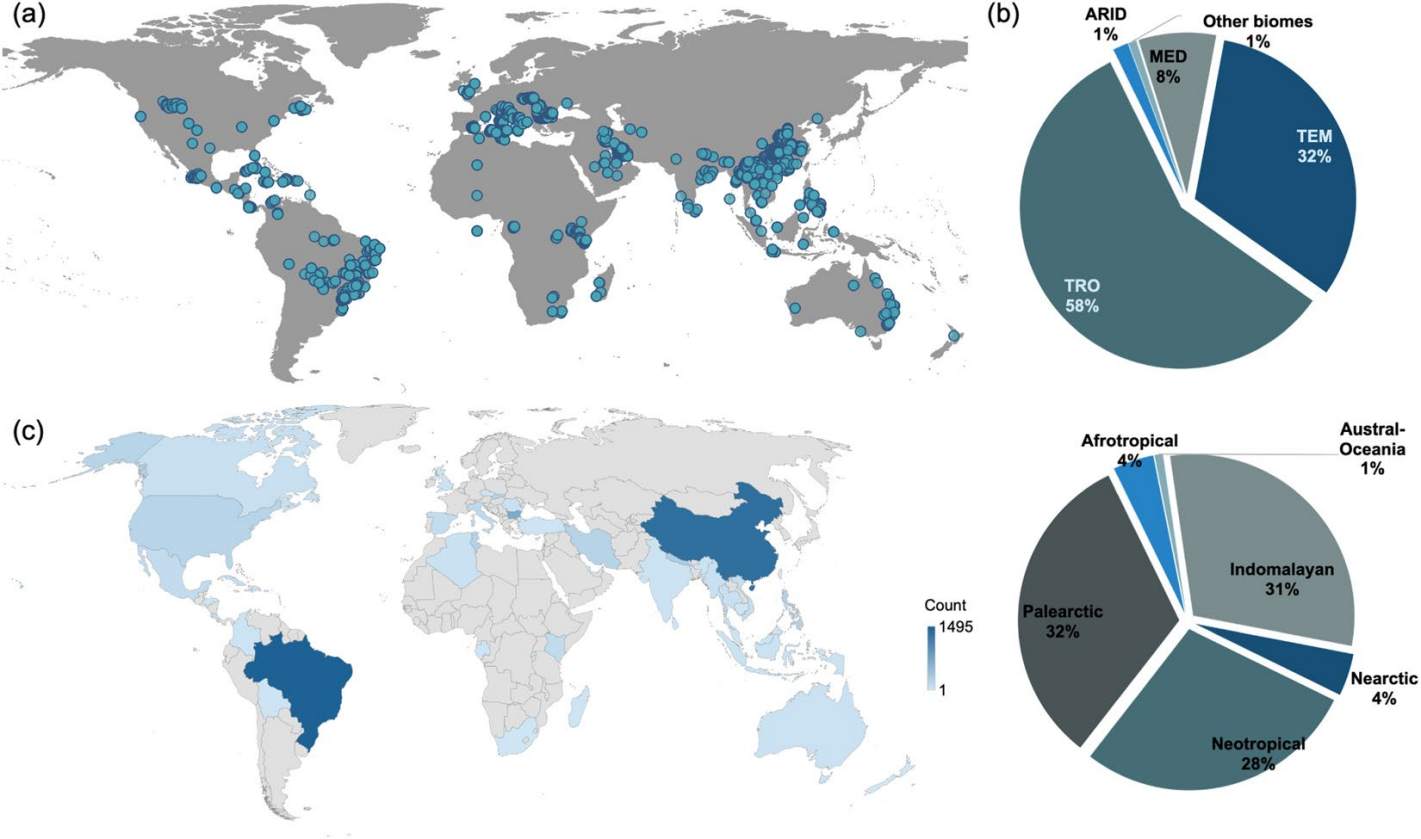
The geographical data turnover of the current database version: (**a**) geographical locations of all bat caves included in the database, (**b**) percent distribution of species occurrence in terms of the biogeographical realm and terrestrial biome, (**c**) country-level turnover.

The second dataset contains the bat cave geographical location (latitude/longitude) and recorded species (Table 2, Fig. 3a). We used the Web of Science and Google Scholar to search online literature, databases, and repositories for published information on cave-dwelling bats from 1990 to 2021. We used the following combination of keywords: (Bat* OR Chiroptera OR Chiroptera fauna*) AND (Diversity OR “Species richness” OR abundance OR distribution OR conservation OR ecology) AND (Cave* OR Cave-dwelling OR Cave-roosting OR underground* OR subterranean OR karst* OR Limestone). We also set a “create alert” in Google Scholar whenever new related papers were published. The data mining process for version 1.0 ended in June 2021. Our search returned 753 papers. We also searched using the Baidu Research engine for Chinese literature and self-archived ResearchGate to maximise search results. To ensure the precision of the datasets included in DarkCideS 1.0, we filtered all published literature to only include those papers or reports with complete species names and geographical records. We contacted corresponding authors with requests to provide us with geographical data when these were missing from their papers or supplementary materials. In the circumstance that we were unable to find the data, and the corresponding author did not respond to our request, that “cave site” was excluded from the database. We converted all species and cave latitude and longitude into WG8 84 decimal degrees with five significant figures. The second dataset of DarkCideS 1.0 contains 6746 georeferenced occurrences for 402 species^11^ from 2002 cave sites (Fig. 3a). Cave sites occur in all continents except Antarctica, with most of the data originating from tropical and temperate biomes (Fig. 3b). We have cave records from 46 countries of which China and Brazil have the highest number of caves recorded (Fig. 3c).

**Table 2:** Metadata of the georeferenced information of cave-dwelling bats and caves.

Data Column	Data type	Data filters
Biogeographical.realm	Nominal	Afrotropical
		Indomalayan
		Austral-Oceania
		Neotropical
		Palearctic
		Nearctic
Biome.classification	Nominal	Deserts & Xeric Shrublands = DES
		Flooded Grasslands & Savannas = FLO
		Mangroves = MAN
		Mediterranean Forests, Woodlands & Scrub = MFWS
		Montane Grasslands & Shrublands = MGS
		Temperate Broadleaf & Mixed Forests = TBMF
		Temperate Conifer Forests = TCF
		Temperate Grasslands, Savannas & Shrublands = TGSS
		Tropical & Subtropical Coniferous Forests = TSCF
		Tropical & Subtropical Dry Broadleaf Forests = TSDB
		Tropical & Subtropical Grasslands, Savannas & Shrublands = TSGS
		Tropical & Subtropical Moist Broadleaf Forests = TSMB
Country.record	Nominal	All countries with records
Latitude	Continuous (WGS 84 in DD)	N/A
Longitude	Continuous (WGS 84 in DD)	N/A

### Cave landscape features and vulnerabilities

**Fig. 4:**
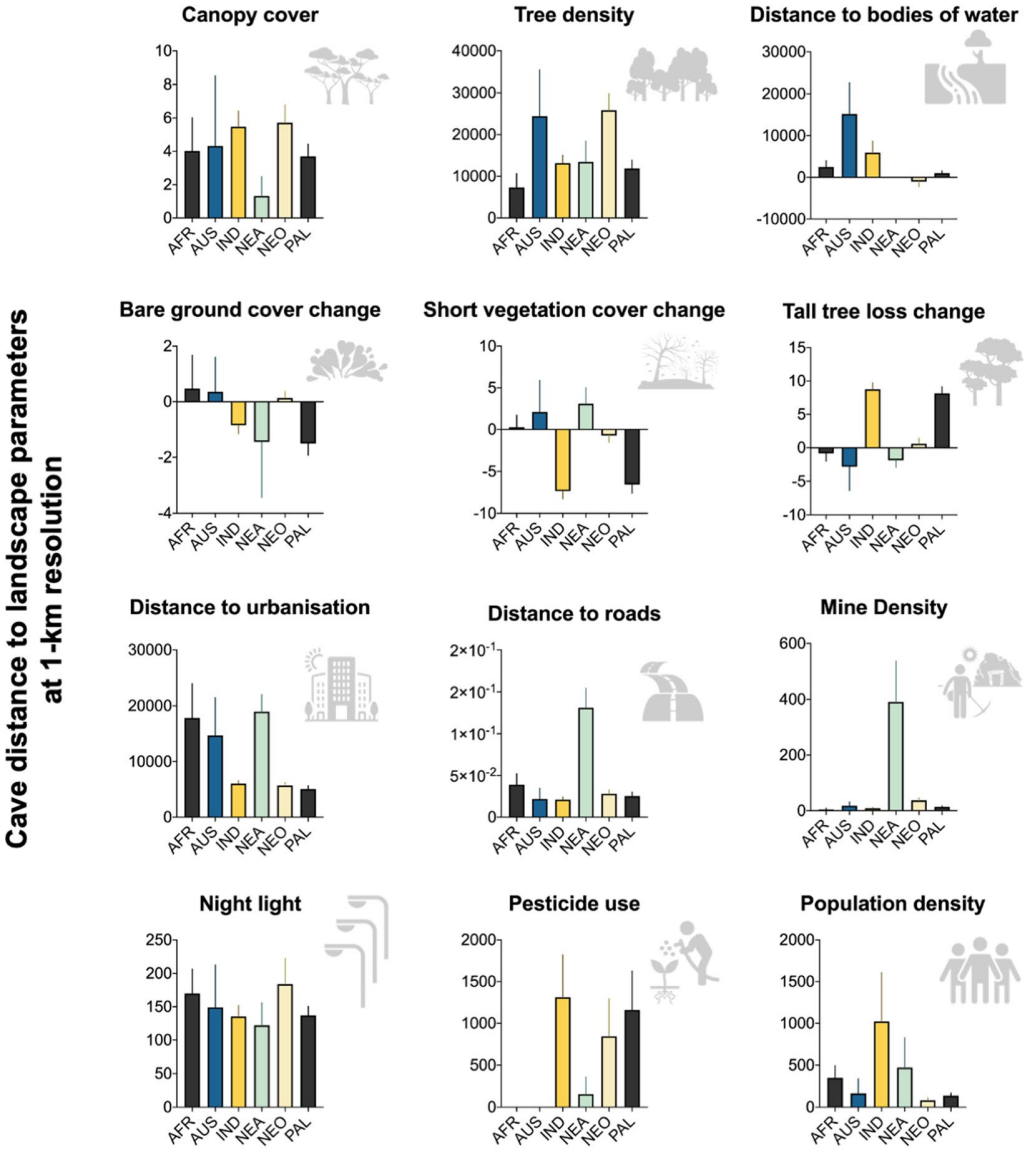
Biogeographical comparison (mean, 95% CI) of landscape parameters at 1-km resolution.

The condition of surface ecosystems and the extent of threats are significant determinants of cave-dwelling bat diversity^11^. Yet, standardising the vulnerability of caves and underground ecosystems from threats on a global scale is challenging^11,14^. To address this, the surface ecosystem was mapped as a proxy to assay cave vulnerability to threats using remotely sensed landscape features. The third dataset included in the database contains the measured land-use and landscape features of the cave surroundings using the georeferenced data from the second dataset (Table 3; Fig. 4). The selected landscape features measurements of the 2002 cave sites were selected based on Tanalgo *et al*.^11^. We included the estimated distance and measures of twelve landscape variables in the database, including canopy cover height^33^, tree density^34^, distance to freshwater bodies^35^, bare ground cover change^36^, short vegetation cover change^36^, tall tree cover change^36^, for vulnerabilities we included distance to urban areas^36^, distance to roads^37^, mine density^38^, night light^39^, relative pesticide exposure^40^, and population density^41,42^. For distance variables, the “distance to feature” tool was used in ArcMap 10.3 and distances were mapped at a 1-km resolution.

**Table 3:** Bat cave distance at 1-km resolution to landscape features included in the current version of the database.

Variables	Variable type	Data Filters	Description	Sources
Biogeographical.realm	Nominal	Afrotropical	N/A	N/A
		Indomalayan	N/A	
		Austral-Oceania	N/A	
		Neotropical	N/A	
		Palearctic	N/A	
		Nearctic	N/A	
Region		All continents entered	N/A	
Country		All countries entered	N/A	
Cave_Name		All cave names entered	N/A	
Latitude	Continuous (WGS84 DD)	N/A	N/A	
Longitude	Continuous (WGS84 DD)	N/A	N/A	
Canopy cover height	Continuous (see source for units) (in 1-km distance resolution)	Canopy.cov	A wall-to-wall, global map of canopy height at 1-km spatial resolution	Simard *et al*.^34^
Tree density		Tree.dens	A spatially continuous map of forest tree density based in global scale.	Crowther *et al*.^35^
Distance to freshwater bodies		Freshwater.dist	A global 3arc-second Water Body Map (G3WBM)	Yamazaki *et al*.^36^
Bare ground cover change		Bareground.change	Continuous global vegetation for tall vegetation ( ≥ 5 m in height; hereafter referred to as tree canopy (TC)) cover, short vegetation (SV) cover and bare ground (BG) cover, at 0.05° × 0.05° spatial resolution	Song *et al*.^37^
Short vegetation cover change		Shortveg.change	Continuous global vegetation for tall vegetation ( ≥ 5 m in height; hereafter referred to as tree canopy (TC)) cover, short vegetation (SV) cover and bare ground (BG) cover, at 0.05° × 0.05° spatial resolution	Song *et al*.^37^
Tall tree cover change		Talltree.change	Continuous global vegetation for tall vegetation ( ≥ 5 m in height; hereafter referred to as tree canopy (TC)) cover, short vegetation (SV) cover and bare ground (BG) cover, at 0.05° × 0.05° spatial resolution	Song *et al*.^37^
Distance to urban areas		Urban.dist	Continuous global vegetation for tall vegetation ( ≥ 5 m in height; hereafter referred to as tree canopy (TC)) cover, short vegetation (SV) cover and bare ground (BG) cover, at 0.05° × 0.05° spatial resolution	Song *et al*.^37^
Distance to roads		Road.dist	A globally harmonised map for road networks and road density at a 5 arcminutes resolution (~8x8km) based on Global Road Inventory Project	Meijer *et al*.^38^
Mine density		Mine.dens	A global distribution of selected critical mineral resources in mines, deposits, districts, and regions	Labay *et al*.^39^
Nightlight		Nightlight	Satellite images of Earth at night based on 2016 cloud-free observations over land mass. The image is divided in to three different resolutions: 0.1 degrees (3600 × 1800), 3 km (13500 × 6750), and 500 m (86400 × 43200).	Earth at Night^40^
Relative pesticide exposure		Pesticide.exp	A database of the 20 most used pesticide active ingredients on 6 dominant crops and 4 aggregated crop classes at 5 arc-min resolution (about 10 km at the equator) projected from 2015 to 2025	Maggi *et al*.^41^
Population density		Pop.dens	Population input data are collated from the 2010 round of Population and Housing Censuses, from 2005 and 2014 data. The input data are extrapolated to produce population estimates for the years 2000, 2005, 2010, 2015, and 2020. GPWv4 is gridded with an output resolution of 30 arc-seconds (approximately 1 km at the equator).	Hughes^42^; SEDAC^43^

### Cave bat parasites and hyperparasites

Parasites, while being among the most diverse modes of life, are often disregarded in conservation strategies^43^. It is well established that parasites affect the stability of food webs and ecosystem health, but hyperparasites have thus far been severely understudied. For future studies on host associations across multiple trophic levels and on the effects of climatic conditions and land-use changes, parasites and hyperparasites are part of our DarkCideS 1.0 database. The fourth dataset lists the parasitic bat flies and their Laboulbeniales fungal hyperparasites associated with cave bats. Data were collected from several sources, including our fieldwork data^36^, Haelewaters *et al*.^44^, and de Groot *et al*.^45^. Bat fly taxonomy followed Dick and Graciolli^46^ and Graciolli and Dick^47^ and fungal taxonomy followed Index Fungorum^48^. In addition to the conspicuous bat flies, bats are host to several other lineages of parasites mites and ticks, lice, fleas, bugs, and earwigs^49,50^. Consequently, the fourth dataset will be expanded on in future versions of DarkCideS with data on these parasitic organisms. A recent call for global collaborations among bat scientists and collaborations to generate multitrophic data of bats, bat flies, and fungi^50^ along with the current DarkCideS 1.0 initiative will contribute to a general understanding of how ecological and life-history traits are correlated with bat parasitism and how host associations may change under changing conditions.

## Data Records

The complete database for global cave-dwelling bats was organised in four main datasets stored in separate Excel workbooks (.csv file format). Each dataset contains unique sequential name IDs that correspond to metadata, variables, and references. All datasets included in the database are available and open-access from Figshare online repository^51^ and through a public website (https://darkcides.org/). The resolution of the publicly available cave and species occurrences were reduced for the protection of caves and to prevent hunting and harvesting. Database users can request high-resolution data of georeferenced species occurrence and cave sites from the corresponding authors. When a substantial dataset is available, all additional datasets will be updated in Figshare.

## Technical Validation

**Fig. 5:**
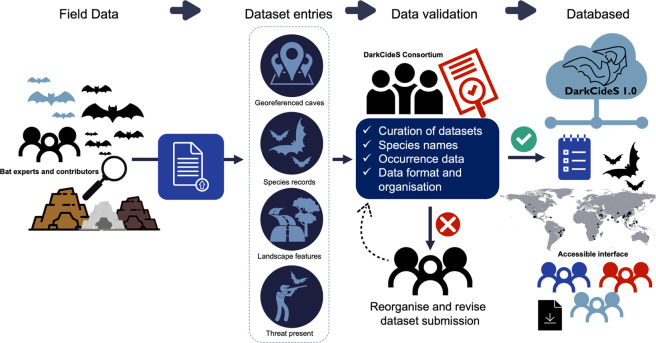
Schematic diagram showing the updating workflow of the database from new data entry. The DarkCideS database aims to be a long-term biodiversity data exchange platform by including new data from fieldwork and assessments. Authors can upload their dataset containing species records, geographical information, and landscape threats on the web page. The corresponding authors will receive new data entries for validation before being merged into the database.

The data included in this database are mainly derived from public, expert-based databases, published material and bat researchers, therefore ensuring the accuracy of the included data. We provided the corresponding reference (when applicable) for each cave record for cross-referencing and data validation purposes. When published “cave datasets” were unclear or lacked detailed information, we communicated with the corresponding authors. We encourage continued contributions to the DarkCideS database as we aim to regularly update the entries for species checklists, traits, geographical locations of caves, and species occurrence data. For species ecological status (e.g., current conservation status, population trends, geopolitical endemism), we will update entries after every IUCN Red List assessment cycle. The database will be updated when new data are contributed and corrected when an error in the data entry is reported to any of the corresponding authors. New entries will be quality screened based on the criteria listed above before adding to the database (Fig. 5). Once an update is made, a release note will be published on the database website. When updating new versions of DarkCideS, we will continue to make available previous releases. Contributors will be included as co-authors when the next version of the database is published. Furthermore, as each cave has a unique ID, additional surveys of other taxa at the same locality can be integrated into the database, to provide a backbone for enhancing our understanding of cave biodiversity through time.

## Usage Notes

All datasets included in DarkCideS are publicly available under a Creative Commons Attribution 4.0 International Public Licences (https://creativecommons.org/licenses/by/4.0/), where users and authors may freely use our datasets, with the condition that the sources are credited and acknowledged, the original license is linked, and any modifications and treatments to our data are indicated in the final work or material.

Although we aim to maximise spatial coverage with datasets from across the globe, we acknowledge that geographical biases inevitably exist^52^. For example, we have multiple datasets from the Palearctic, Indomalayan, and Neotropical realms, whereas very little data, originated from the Afrotropical region (see Fig. 3). We also encountered similar coverage bias in country-level data richness. For example, Indonesia is one of the most diverse countries for estimated cave-dwelling bat species richness^11^, but a very small number of species were included in the current version of the database. The database is intended as a long-term data-sharing platform, and we hope to fill these gaps in the next versions of the database. Further data and better coverage will provide a better index for regional prioritisation in addition to further research on bat diversity patterns and threats.

### Consortia authorship

The DarkCideS database is a continuous project. To promote global collaboration and equitability, all present and future members of the DarkCideS initiative and consortia (https://darkcides.org/our-team/) will be considered bona fide authors of the current and future versions of the database.

## Data Availability

No code was used to generate the data presented in this data paper.
